# Troxerutin Attenuates Enhancement of Hepatic Gluconeogenesis by Inhibiting NOD Activation-Mediated Inflammation in High-Fat Diet-Treated Mice

**DOI:** 10.3390/ijms18010031

**Published:** 2016-12-25

**Authors:** Zifeng Zhang, Xin Wang, Guihong Zheng, Qun Shan, Jun Lu, Shaohua Fan, Chunhui Sun, Dongmei Wu, Cheng Zhang, Weitong Su, Junwen Sui, Yuanlin Zheng

**Affiliations:** 1 Key Laboratory for Biotechnology on Medicinal Plants of Jiangsu Province, School of Life Science, Jiangsu Normal University, Xuzhou 221116, China; zhangzifengsuper@jsnu.edu.cn (Z.Z.); xznkywx@163.com (X.W.); 6020030110@jsnu.edu.cn (G.Z.); shanp@jsnu.edu.cn (Q.S.); lu-jun75@163.com (J.L.); fshfly@jsnu.edu.cn (S.F.); chunhuisun@jsnu.edu.cn (C.S.); wdm8610@163.com (D.W.); 2020160758@jsnu.edu.cn (C.Z.); 3020143834@jsnu.edu.cn (W.S.); suijunwen16@163.com (J.S.); 2 Key Laboratory of Biology and Genetic Improvement of Sweetpotato, Ministry of Agriculture, Jiangsu Xuzhou Sweetpotato Research Center, Xuzhou Institute of Agricultural Sciences in Xuhuai Distric, Xuzhou 221131, China

**Keywords:** troxerutin, hepatic gluconeogenesis, fasting hyperglycemia, endoplasmic reticulum stress, nucleotide oligomerization domain protein, inflammation

## Abstract

Recent evidence suggests that troxerutin, a trihydroxyethylated derivative of natural bioflavonoid rutin, exhibits beneficial effects on diabetes-related symptoms. Here we investigated the effects of troxerutin on the enhancement of hepatic gluconeogenesis in high-fat diet (HFD)-treated mice and the mechanisms underlying these effects. Mice were divided into four groups: Control group, HFD group, HFD + Troxerutin group, and Troxerutin group. Troxerutin was treated by daily oral administration at doses of 150 mg/kg/day for 20 weeks. Tauroursodeoxycholic acid (TUDCA) was used to inhibit endoplasmic reticulum stress (ER stress). Our results showed that troxerutin effectively improved obesity and related metabolic parameters, and liver injuries in HFD-treated mouse. Furthermore, troxerutin significantly attenuated enhancement of hepatic gluconeogenesis in HFD-fed mouse. Moreover, troxerutin notably suppressed nuclear factor-κB (NF-κB) p65 transcriptional activation and release of inflammatory cytokines in HFD-treated mouse livers. Mechanismly, troxerutin dramatically decreased Nucleotide oligomerization domain (NOD) expression, as well as interaction between NOD1/2 with interacting protein-2 (RIP2), by abating oxidative stress-induced ER stress in HFD-treated mouse livers, which was confirmed by TUDCA treatment. These improvement effects of troxerutin on hepatic glucose disorders might be mediated by its anti-obesity effect. In conclusion, troxerutin markedly diminished HFD-induced enhancement of hepatic gluconeogenesis via its inhibitory effects on ER stress-mediated NOD activation and consequent inflammation, which might be mediated by its anti-obesity effect.

## 1. Introduction

Over the past two decades, it has been increasingly appreciated that low-grade inflammation plays a crucial role in type 2 diabetes (T2D) etiology and progression [1]. Excess caloric consumption and obesity are suggested to cause a sustained systemic inflammation, which impairs the insulin-secretory capacity of pancreas and insulin sensitivity of peripheral tissues, including fat, muscle, and liver, thereby contributing to the development of T2D [2,3]. Though the importance of inflammation for developing T2D has been well established, the triggering mechanisms responsible for inflammation during excess caloric consumption and obesity are still not well understood.

Nucleotide oligomerization domain (NOD) proteins including NOD1 and NOD2, which belong to a class of intracellular pattern recognition receptors, sense invading bacteria by recognizing intracellular fragments of bacterial peptidoglycan (pathogen-associated molecular patterns, PAMPs), thereby activating innate immune response to clear those pathogens [4,5]. In addition to monitoring PAMPs, NOD1 and NOD2 are recently reported to detect endogenous molecules derived from tissue/cellular injuries called damage-associated molecular patterns (DAMPs) and thereby induce proinflammatory responses [6,7]. In T2D, tissue injuries mediated by oxidative stress cause the release of DAMPs, resulting in pattern recognition receptor activation and consequent inflammatory responses [8,9,10]. It is well established that oxidative stress is an important cause of ER stress [11,12]. However, whether ER stress activates NOD to trigger inflammation during T2D has never been investigated.

Troxerutin, known as vitamin P4, is a trihydroxyethylated derivative of natural bioflavonoid rutin, which is widely distributed in tea, coffee, cereal grains, and a variety of fruits and vegetable [13,14,15]. Troxerutin exhibits multiple biological activities, such as antioxidant, anti-inflammatory and hepatoprotective activities [16,17,18]. It has been demonstrated that troxerutin effectively improves diabetes-related symptoms, such as obesity, hyperlipidemia and insulin resistance [18,19,20]. Our previous work shows that troxerutin protects against inflammation-mediated tissue injuries under diverse pathological conditions [13,17]. Nevertheless, the protective effects of troxerutin on inflammation-mediated development of T2D and its protective mechanisms have yet to be elucidated. Hyperglycemia, the hallmark metabolic abnormality of T2D, is a major risk factor for the development and progression of diabetes complications such as vascular disease, neuropathy, and nephropathy [21,22]. It is well established that hepatic gluconeogenesis is aberrantly enhanced in T2D, which plays a predominant role in elevating hepatic glucose production, leading to fasting hyperglycemia during T2D [23,24]. Substantial evidence demonstrated that inflammation is an important cause of up-regulation of hepatic gluconeogenesis and consequent fasting hyperglycemia [25,26]. During last several decades, many therapies, such as lifestyle-directed interventions, insulin, and metformin, have been developed to control the glucose levels. Recent evidence indicates that naturally-occurring polyphenols exhibit evident blood glucose-lowering effects, which have a powerful beneficial effect on treatment of diabetes [27,28]. Thus, in this study, we postulated that troxerutin might inhibit hepatic inflammatory responses by attenuating ER stress-mediated NOD activation, thereby diminishing hepatic gluconeogenesis and consequent hyperglycemia in high-fat diet (HFD)-induced diabetic mice. This study was designed to address these issues.

## 2. Results

### 2.1. Troxerutin Improves Obesity and Related Metabolic Parameters, and Liver Injuries in HFD-Treated Mice

**Figure 1:**
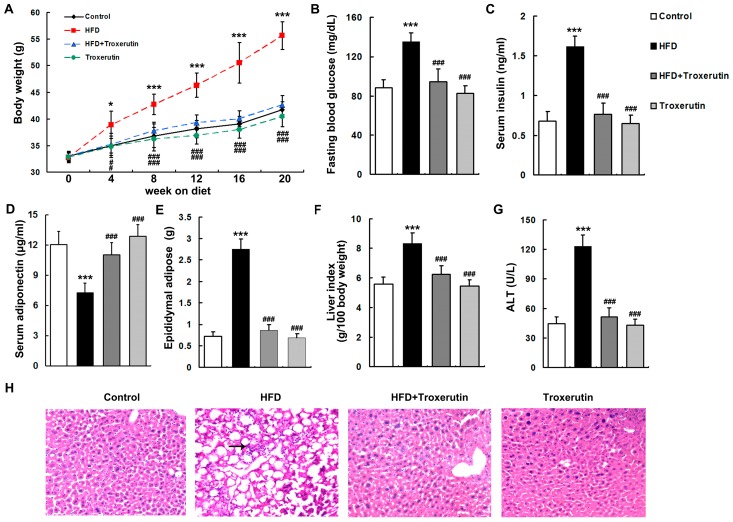
Troxerutin improves obesity and related metabolic parameters, and liver injuries in high-fat diet (HFD)-treated mouse (*n* = 5). (**A**) Total body weight in all treated groups; (**B**) The levels of fasting blood glucose in all treated groups; (**C**) The levels of serum insulin in all treated groups; (**D**) The levels of serum adiponectin in all treated groups; (**E**) The levels of epididymal adipose tissue masses in all treated groups; (**F**) The levels of liver index in all treated groups; (**G**) Serum alanine aminotransferase (ALT) activities in all treated groups; (**H**) H&E staining of liver sections, 200× magnification. Inflammatory cells are indicated by black arrow. All of the values are expressed as the mean ± SD. * *p* < 0.05, *** *p* < 0.001 vs. the control group; ### *p* < 0.001 vs. the HFD group.

After four weeks of treatment, HFD-fed mice developed significant obesity (Figure 1A). Furthermore, HFD feeding caused strikingly increased levels of obesity-related metabolic parameters including fasting blood glucose, serum insulin, epididymal adipose tissue masses, and liver indices, and decreased serum adiponectin level in mice (Figure 1B–F). Interestingly, troxerutin effectively lowered body weight and obesity-related metabolic parameters in HFD-treated mice (Figure 1A–F). There were no evident differences in body weight and obesity-related metabolic parameters among the HFD + Troxerutin, Troxerutin and Control groups.

An evident liver injury, as evidenced by significantly elevated serum alanine aminotransferase (ALT) level, was observed in HFD-treated mice (Figure 1G). Hematoxylin and eosin (H&E) staining indicated that HFD feeding resulted in hepatocyte hypertrophy and vacuolization and inflammatory cell infiltration in the mouse livers (Figure 1H). Interestingly, oral administration of troxerutin notably inhibited those liver injuries in HFD-treated mice (Figure 1G,H). No significant differences in liver injuries were found among the HFD + Troxerutin, Troxerutin and Control groups.

These results indicate that troxerutin improves obesity and related metabolic parameters, and liver injuries in HFD-treated mouse.

### 2.2. Troxerutin Attenuates Hepatic Gluconeogenesis in HFD-Treated Mice

**Figure 2:**
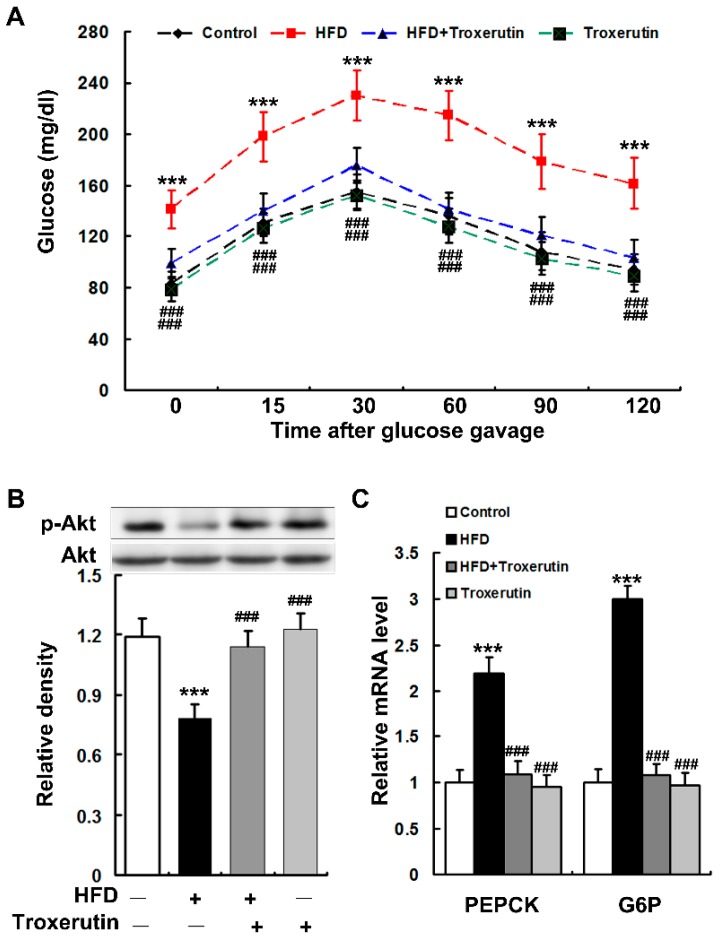
Troxerutin attenuates hepatic gluconeogenesis in HFD-treated mice. (**A**) Data of glucose tolerance tests in different treatment groups. (*n* = 5); (**B**) Immunoblotting and densitometry of p-Akt (serine 473) in mouse livers (*n* = 3); (**C**) The mRNA levels of gluconeogenic genes in mouse livers (*n* = 3). All of the values are expressed as the mean ± SD. *** *p* < 0.001 vs. the control group; ### *p* < 0.001 vs. the HFD group.

HFD feeding markedly impaired glucose intolerance in mice as evidenced by a diminished ability to lower their blood glucose (Figure 2A). A dramatically reduced phosphorylation of Akt (serine 473) was observed in the livers of HFD-treated mice (Figure 2B), indicating an impairment of insulin signaling. Moreover, HFD feeding notably enhanced gluconeogenesis as indicated by the augmented mRNA levels of phosphoenolpyruvate carboxykinase (PEPCK) and glucose-6-phosphatase (G6P) in the mouse livers (Figure 2C). Interestingly, troxerutin dramatically restored glucose intolerance and insulin signaling, and diminished hepatic gluconeogenesis in HFD-treated mice (Figure 2). There were no significant differences in glucose intolerance, insulin signaling and hepatic gluconeogenesis among the HFD + Troxerutin, Troxerutin and the vehicle control groups.

These results suggest that troxerutin restores hepatic insulin signaling to diminish gluconeogenesis in HFD-treated mouse.

### 2.3. Troxerutin Inhibits Inflammatory Response in HFD-Treated Mouse Livers

**Figure 3:**
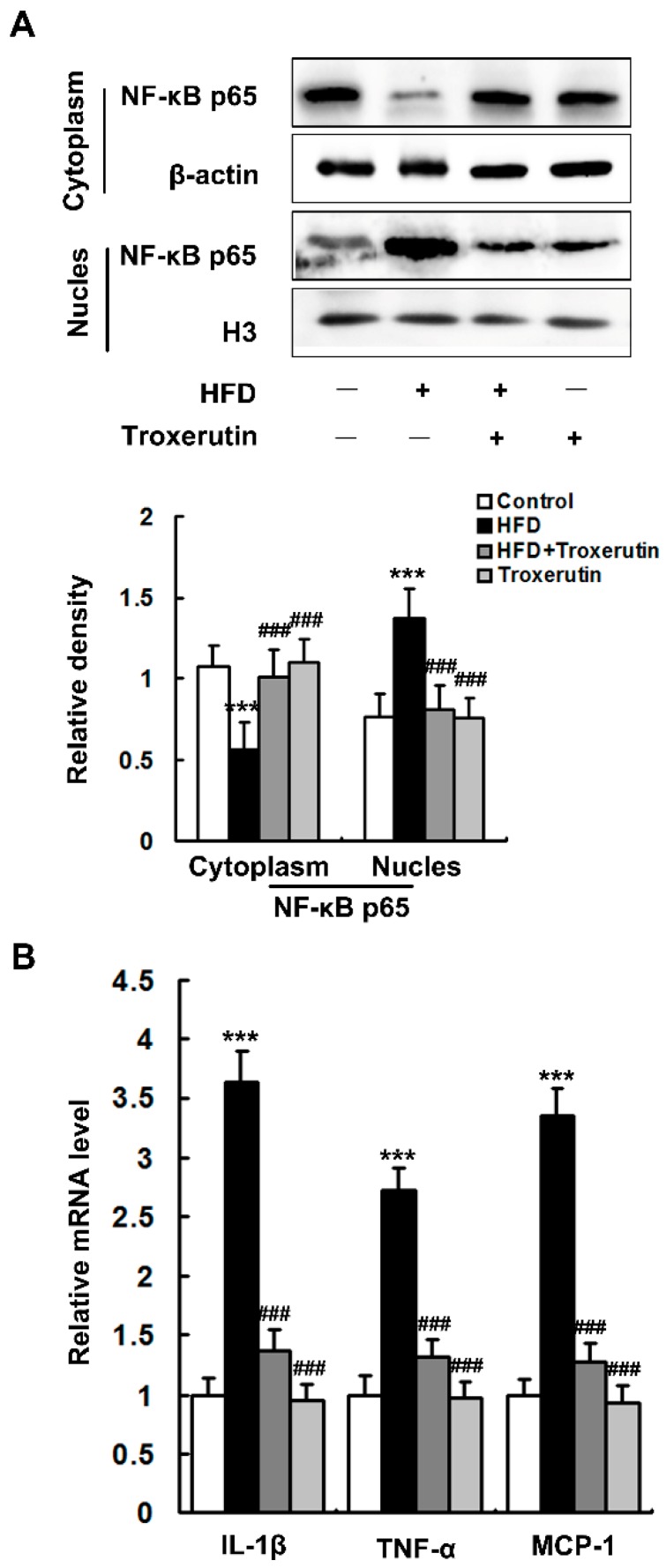
Troxerutin inhibits inflammatory response in the HFD-treated mouse livers (*n* = 3). (**A**) Immunoblotting and densitometry of nuclear and cytoplasmic NF-κB p65 in mouse livers; (**B**) The mRNA level of inflammation-related genes in mouse livers. All of the values are expressed as the mean ± SD. *** *p* < 0.001 vs. the control group; ### *p* < 0.001 vs. the HFD group.

HFD feeding dramatically increased nuclear and decreased cytoplasmic nuclear factor-κB (NF-κB) p65 subunit localization in the mouse livers (Figure 3A), indicating that NF-κB nuclear translocation were promoted by HFD treatment. Moreover, HFD feeding significantly augmented the mRNA expression levels of NF-κB target genes in the mouse livers, such as *interleukin-1β* (IL-1β), *tumor necrosis factor-α* (TNF-α) and *monocyte chemoattractant protein-1* (MCP-1) (Figure 3B). Interestingly, troxerutin remarkably inhibits the nuclear translocation of NF-κB p65, as well as the expressions of its target genes, in the livers of HFD-treated mice (Figure 3). No significant differences in NF-κB p65 nuclear translocation and its target gene expression were found among the HFD + Troxerutin, Troxerutin and Control groups.

In combination with the abovementioned results of H&E staining (Figure 1G), these findings indicate that troxerutin protects against HFD-induced liver inflammatory response in mice.

### 2.4. Troxerutin Attenuates Oxidative Stress in HFD-Treated Mouse Livers

**Figure 4:**
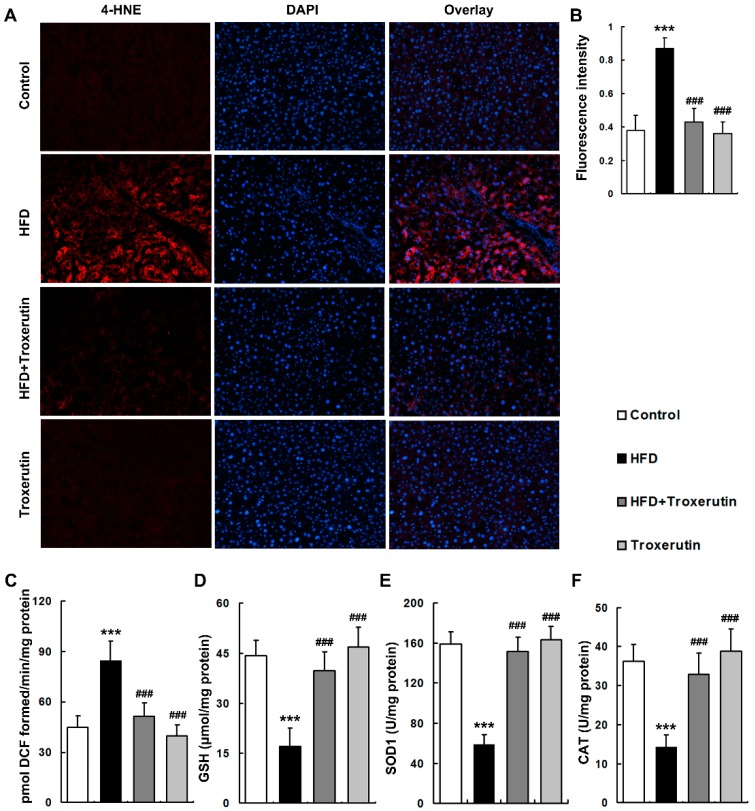
Troxerutin suppresses oxidative stress in HFD-treated mouse livers (*n* = 5). (**A**) 4-HNE immunofluorescence staining, 200× magnification; (**B**) 4-HNE fluorescence intensity was measured as the mean OD value; (**C**) ROS productions in mouse livers; (**D**) GSH contents in mouse livers; (**E**) SOD1 activities in mouse livers; (**F**) CAT activities in mouse livers. All of the values are expressed as the mean ± SD. *** *p* < 0.001 vs. the control group; ### *p* < 0.001 vs. the HFD group.

The levels of oxidative stress markers, including reactive oxygen species (ROS) and 4-hydroxynonenal (4-HNE, a marker of lipid peroxidation), were markedly elevated in the livers of HFD-treated mice (Figure 4A–C). Moreover, HFD feeding remarkably depressed glutathione (GSH) content and activities of antioxidant enzymes including superoxide dismutases 1 (SOD1) and catalase (CAT) in the mouse livers (Figure 4D–F), suggesting a notable impairment of hepatic reducing potential. Interestingly, troxerutin dramatically decreased ROS and 4-HNE levels, and restored GSH content and these antioxidant enzymes activities in the livers of HFD-treated mice (Figure 4). There were no significant differences in the levels of oxidative stress markers and reducing potential among the HFD + Troxerutin, Troxerutin and Control groups.

These results indicate that troxerutin blocks oxidative stress in the HFD-treated mouse livers.

### 2.5. Troxerutin Abates ER Stress in HFD-Treated Mouse Livers

**Figure 5:**
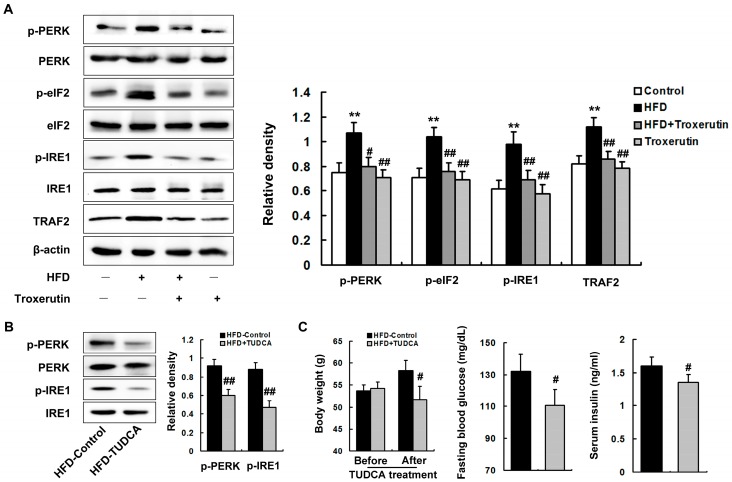
Troxerutin abates ER stress in HFD-treated mouse livers. (**A**) Immunoblotting and densitometry of ER stress-relative proteins in mouse livers (*n* = 3); (**B**) Immunoblotting and densitometry of ER stress markers in mouse livers (*n* = 3); (**C**) Total body weight, serum insulin, and fasting blood glucose in mouse livers (*n* = 5); All of the values are expressed as the mean ± SD. ** *p* < 0.01 vs. the control group; # *p* < 0.05, ## *p* < 0.01 vs. the HFD group.

The unfolded protein response (UPR) was largely activated, characterized by the up-regulations of phosphorylated pancreatic endoplasmic reticulum resident kinase (p-PERK) (Thr980), phosphorylated eukaryotic translation initiation factor (p-eIF2α) (Ser51) and phosphorylated inositol-requiring 1 (p-IRE1) (Ser724) protein levels in the livers of the HFD-treated mice (Figure 5A). Furthermore, HFD feeding significantly up-regulated the protein level of TNF receptor-associated factor 2 (TRAF2), a downstream protein of IRE1 signaling, in the livers of the HFD-treated mice (Figure 5A), indicating the occurrence of ER stress.

ER stress is critical to the development of a variety of diseases, including diabetes [12,29]. In this study, we blocked ER stress using tauroursodeoxycholic acid (TUDCA, a well-known inhibitor of ER stress). We found that TUDCA observably inhibited ER stress in the livers of the HFD-treated mice, characterized by the down-regulations of p-PERK (Thr980) and p-IRE1 (Ser724) protein levels (Figure 5B). Furthermore, TUDCA effectively deceased body weight, fasting blood glucose, and serum insulin levels in HFD-treated mice (Figure 5C), demonstrating an important role for ER stress in enhancement of hepatic gluconeogenesis-mediated fasting hyperglycemia in HFD-treated mouse.

Interestingly, troxerutin treatment markedly diminished the protein levels of p-PERK (Thr980), p-eIF2α (Ser51), p-IRE1 (Ser724) and TRAF2 in the livers of the HFD-treated mice (Figure 5A). No significant differences in ER stress were found among the HFD + Troxerutin, Troxerutin and the vehicle control groups.

These results reveal that troxerutin abates ER stress in the HFD-treated mouse livers, which may attenuate fasting hyperglycemia.

### 2.6. Troxerutin Depresses NOD Activation in HFD-Treated Mouse Livers

**Figure 6:**
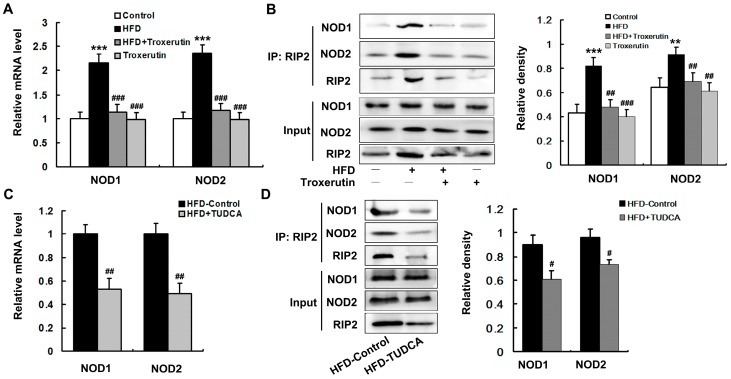
Troxerutin depresses NOD activation in HFD-treated mouse livers (*n* = 3). (**A**,**C**) show the mRNA levels of NOD1 and NOD2 in mouse livers; (**B**,**D**) show the immunoblotting and densitometry of NOD1 and NOD2 after immunoprecipitation, using RIP2 antibody in mouse livers. All of the values are expressed as the mean ± SD. ** *p* < 0.01, *** *p* < 0.001 vs. the control group; # *p* < 0.05, ## *p* < 0.01, ### *p* < 0.001 vs. the HFD group.

HFD caused a marked enhancement of *NOD* expression, including *NOD1* and *NOD2*, in mouse livers (Figure 6A). To determine the interaction between NOD1/2 with receptor interacting protein-2 (RIP2), we used RIP2 antibody to immunoprecipitate RIP2 and immunoblotted for NOD1 and NOD2. The results showed that the association of RIP2 with NOD1/2 was markedly enhanced in the livers of the HFD-treated mice (Figure 6B). Furthermore, we found that *NOD* expression and association of RIP2 with NOD1/2 were dramatically blunted by TUDCA treatment in the HFD-fed mouse livers (Figure 6C,D). These findings suggest that NOD activation is mediated by ER stress in the HFD-treated mouse livers.

Interestingly, troxerutin dramatically decreased *NOD* expression, as well as interaction between NOD1/2 with RIP2 in the livers of the HFD-treated mice (Figure 6A,B). There were no significant differences in *NOD* expression and association of RIP2 with NOD1/2 among the HFD + Troxerutin, Troxerutin and control groups.

Collectively, these results reveal that troxerutin depresses ER stress-mediated NOD activation in HFD-treated mouse livers.

## 3. Discussion

Substantial evidence has demonstrated that lowering of glucose levels have a potent beneficial effect on T2D complications [30,31]. Therefore, management of hyperglycemia takes center stage in current medical therapy for T2D. It is well established that hepatic gluconeogenesis plays a pivotal role in excessive hepatic glucose production and consequent fasting hyperglycemia during T2D [23,24]. In the present study, we revealed that troxerutin effectively ameliorated enhancement of hepatic gluconeogenesis via blocking ER stress-mediated NOD activation and the consequent inflammatory response, thereby improving fasting hyperglycemia in HFD-treated mouse, which might be mediated by its anti-obesity effect. This study provides novel mechanistic insights into the etiology of hyperglycemia and the glucose-lowering effect of natural products.

Chronic inflammation drives the dysregulation of signaling pathways that cause various conditions associated with T2D [2,32]. Peripheral tissue inflammation, particularly fat and liver inflammation, is a well-established cause of T2D and its complications [25,26]. It is suggested that troxerutin exhibits anti-inflammatory effect under diverse liver-injury conditions [13,17]. The present study showed that troxerutin significantly inhibited the inflammatory response in HFD-treated mouse livers. Our work suggested that troxerutin might effectively improve T2D-related symptoms including fasting hyperglycemia through an anti-inflammatory mechanism. Substantial evidence suggests that liver inflammation, which impairs hepatic insulin signaling, increases hepatic gluconeogenesis, leading to fasting hyperglycemia in T2D [25,26,33]. This study showed that troxerutin markedly improved hepatic insulin signaling to attenuate hepatic gluconeogenesis. Thus, our work further revealed that troxerutin evidently suppressed enhancement of hepatic gluconeogenesis to ameliorate fasting hyperglycemia by abating inflammation in HFD-treated mouse.

NOD activation has been implicated in the pathogenesis of a variety of inflammatory diseases including diabetes mellitus [4,5,6]. Recently, NOD activation has been demonstrated to provoke insulin resistance by triggering inflammatory response in peripheral tissue such as in liver, which contribute to the development of T2D [34,35,36,37]. In this study, HFD caused a marked activation of NOD in mouse livers, suggesting that NOD activation-mediated inflammation might play a role in HFD-induced pathological features of T2D including enhancement of hepatic gluconeogenesis. Recently, natural plant products such as natural polyphenolic compound have been demonstrated to exhibit their beneficial effects on various inflammatory diseases by suppressing NOD signaling [38,39]. In this study, our results showed that troxerutin markedly depressed NOD activation in HFD-treated mouse livers. Consistently, our findings revealed that troxerutin improved symptoms of T2D including enhancement of hepatic gluconeogenesis through a mechanism involving inhibition of NOD signaling.

It is well established ER stress is a major contributor to inflammatory diseases including T2D. In the present study, our results showed that troxerutin notably blunted HFD-provoked ER stress in mouse livers. Furthermore, HFD-induced obesity and fasting hyperglycemia were evidently improved by TUDCA treatment (ER stress inhibitor). Therefore, our results suggested that troxerutin might ameliorate the enhancement of hepatic gluconeogenesis-mediated fasting hyperglycemia by abating ER stress in HFD-treated mouse. A recent study reveals that ER stress induces inflammation through triggering NOD signaling during tissue damage and microbial infection [40]. It is well established that excess caloric consumption and obesity chronically elevating the circulating levels of bacterial elements, which may stimulate NOD signaling in peripheral tissues, especially in liver [41,42]. Moreover, increasing evidence suggests that DAMPs activate NOD signaling-mediated proinflammatory responses under various conditions of tissue injury and disease, including T2D [6,7]. At the same time, both circulating bacterial elements and DAMPs derived from tissue/cellular injuries may promote ER stress in peripheral tissues during T2D [43,44], indicating that PAMPs and DAMPs may activate NOD signaling by triggering ER stress. In this study, TUDCA treatment dramatically restrained NOD activation in HFD-treated mouse livers, as well as troxerutin treatment. Consistently, our results revealed that troxerutin blocked HFD-induced inflammation by depressing ER stress-mediated NOD activation in mouse livers. It is well demonstrated that oxidative stress is the most common pathologic condition linking diverse mechanisms for the pathogenesis of T2D. An accumulating body of evidence reveals that natural plant products exhibit their significant beneficial effects on T2D and its complications via their antioxidant activities [45,46]. Consistent with our previous report [18], this study showed that troxerutin dramatically suppressed oxidative stress in the livers of HFD-treated mouse. Our findings indicated that troxerutin effectively improved enhancement of hepatic gluconeogenesis and consequent fasting hyperglycemia by abating oxidative stress. Substantial evidence shows that oxidative stress causes oxidative damage to proteins, endoplasmic reticulum membrane and proteasome components, leading to ER stress [14,47,48]. Thus, this study suggested that troxerutin inhibited ER stress via abating oxidative stress in the livers of HFD-treated mouse.

In obesity, excessive accumulation of adipose tissue leads to aberrant adipokine secretion, which plays a critical role in the development of various obesity-related metabolic disorders such as insulin resistance and ectopic lipid accumulation [49]. It is well established that obesity largely decreases the level of circulating adiponectin, the most abundant adipokine, contributing to the disorders of glucose and lipid metabolism in liver [50,51]. Increasing evidence suggests that natural plant polyphenols improve obesity-induced abnormalities of glucose and lipid metabolism in liver by elevating the level of circulating adiponectin [52,53]. It is reported that troxerutin exhibits an anti-obesity effect in diet-induced diabetes mice [18,54]. In the present study, our results showed that troxerutin markedly ameliorated obesity and excessive accumulation of adipose tissue, and increased circulating adiponectin in HFD-treated mice. Consistent with these studies, our findings indicated that troxerutin might improve hepatic glucose disorders, including the enhancement of gluconeogenesis via its anti-obesity effect and consequent elevation of circulating adiponectin in HFD-treated mouse. The role of anti-obesity effect of troxerutin in the improvement of hepatic glucose disorders needs to be studied further.

## 4. Materials and Methods

### 4.1. Reagents

Troxerutin (>99% purity, 3′,4′,7-Tris [*O*-(2-hydroxyethyl)] rutin; CAS NO. 7085-55-4, EINECS NO. 230-389-4; FORMULA: C_33_H_42_O_19_; MOL WT: 742.68) was obtained from Baoji Fangsheng Biotechnology Co., Ltd (Baoji, China). Normal diet (15.9 kJ/g, 10% of energy as fat, 70% of energy as carbohydrates and 20% of energy as protein; D12450B) and HFD diet (21.9 kJ/g, 60% of energy as fat, 20% of energy as protein, 20% of energy as carbohydrate; D12492) were purchased from Research Diets (New Brunswick, NJ, USA).

Antibody sources: rabbit anti-phospho-Akt (Ser473), rabbit anti-total-Akt, rabbit anti-phospho-PERK (Thr980), rabbit anti-total-PERK, rabbit anti-total-eIF2α, rabbit anti-TRAF2, rabbit anti-NF-κB p65, rabbit anti-histone H3 antibodies and HRP-conjugated anti-rabbit antibodies from Cell Signaling Technology (Beverly, MA, USA); rabbit anti-phospho-eIF2α (Ser51), rabbit anti-phopho-IRE1 (Ser724), rabbit anti-total-IRE1 and rabbit anti-NOD1 antibodies from Abcam (Cambridge, UK); rat anti-NOD2 antibody from eBioscience (San Diego, CA, USA); rabbit anti-RIP2, HRP-conjugated anti-mouse and HRP-conjugated anti-rat antibodies from Santa Cruz Biotechnology (Santa Cruz, CA, USA); mouse anti-β-actin antibody from Chemicon (Temecula, CA, USA); rabbit anti-4-HNE antibody from Alpha Diagnostics (San Antonio, TX, USA); Texas Red-conjugated anti-rabbit antibody was from Vector Laboratories (Burlingame, CA, USA).

Other reagents were obtained from the following sources: TUDCA from Calbiochem (San Diego, CA, USA); hematoxylin and eosin from Sigma-Aldrich (St. Louis, MO, USA); nuclear/cytoplasmic isolation kit and bicinchoninic acid assay kit from Pierce Biotechnology (Rockford, IL, USA); ALT assay kit from the Jiancheng Institute of Biotechnology (Nanjing, China); mouse insulin enzyme-linked immunosorbent assay (ELISA) kit from ALPCO Diagnostics (Windham, NH, USA); adiponectin ELISA kit from Abcam (Cambridge, UK); GSH assay kit from Cayman Chemical (Ann Arbor, MI, USA); 20× LumiGLO^®^ Reagent and 20× peroxide from Cell Signaling Technology (Beverly, MA, USA); Trizol reagent from Invitrogen (Carlsbad, CA, USA); Moloney murine leukemia virus reverse transcriptase, random primers, and SYBR premix Ex TaqII from Takara (Dalian, China).

### 4.2. Animals and Treatment

All of the experimental protocols and euthanasia procedures were approved by the Institutional Animal Care and Use Committee of Jiangsu Normal University (Permit Number: 15-0216; 22 September 2015). Male ICR mice (eight weeks old) were purchased from a Branch of the National Rodent Breeder Center (Shanghai, China). Mice were maintained at constant temperature (23 ± 1 °C) and humidity (60%), with food and drinking water ad libitum, and were kept on a 12 h light/dark cycle. After one week of acclimatization to the laboratory conditions, mice were randomly divided into four groups: Control group, HFD group, HFD + Troxerutin group, and Troxerutin group, and received the following treatments for 20 weeks: Mice in the Control group and the Troxerutin group were fed a normal diet. Mice in the HFD group and the HFD + Troxerutin group were fed a HFD.

#### 4.2.1. Troxerutin Treatment

The mice of HFD + Troxerutin group and Troxerutin group were administered troxerutin in distilled water orally at the dose of 150 mg/kg/day. An equal volume of distilled water was given to the mice in the Control group, and HFD group by daily oral gavage. The troxerutin dosage used in this study was according to our previous work [18].

#### 4.2.2. TUDCA Treatment

Eighteen weeks after HFD feeding, some mice of the HFD group were divided into two subgroups. TUDCA, an ER stress inhibitor, solubilized in phosphate buffered saline (PBS), was given to one subgroup (HFD + TUDCA group) during two weeks by daily intraperitoneal (i.p.) injections at the dose of 200 mg/kg/day, and another subgroup (HFD-control group) received daily i.p. injections of an equal volume of PBS.

After 20 weeks of treatment, overnight fasted mice were deeply anaesthetized and sacrificed, and the blood and liver were immediately collected for experiments or stored at −70 °C for later use.

### 4.3. Glucose Tolerance Test

After 19 weeks of HFD treatment, glucose tolerance tests were performed as described in our previous work [47]. Overnight fasted mice were administered glucose (2 g of glucose per kg of body weight) orally. Blood samples were taken by tail venipuncture immediately before (0 min) and after (15, 30, 60, 90, and 120 min) oral administration of glucose. Blood glucose concentrations were measured with an Ascensia Elite glucose meter (Bayer Corporation, Mishawaka, IN, USA).

### 4.4. Liver Slice Collection and Histopathological Analysis

Liver slice collection and hematoxylin-eosin staining were performed as previously described [13,18]. The liver sections were stained with hematoxylin-eosin, and were examined by an expert liver pathologist who was blinded to the treatment groups.

### 4.5. Immunofluorescence Staining

Immunofluorescence staining of cryo-fixed liver sections was performed as described previously [18]. The liver sections were incubated with rabbit anti-HNE antibody (1:100) overnight at 4 °C. The liver sections were rinsed in phosphate buffered saline and then the following secondary antibodies were used: Texas Red-conjugated anti-rabbit IgG (1:200).

### 4.6. Tissue Homogenates

The preparation of liver homogenates were performed as described in our previous work [13,18]. The protein concentration in the supernatants were determined using the bicinchoninic acid assay kit following the manufacturer’s instructions.

### 4.7. Biochemical Analyses

The serum ALT activity was measured by spectrophotometric methods using a kit according to the manufacturer’s instructions. Fasting blood glucose levels were determined with an Ascensia Elite glucose meter. Serum insulin and adiponectin levels were determined with the enzyme-linked immunosorbent assay kits following the manufacturer’s instructions.

### 4.8. ROS Assay

ROS level was assayed as previously described, which was based on the oxidation of 2′,7′-dichlorodihydrofluorescein diacetate (H2-DCF-DA) to 2′,7′-dichlorofluorescein (DCF) [13,18]. ROS production was presented as pmol 2′,7′-dichlorofluorescein formed/min/mg protein.

### 4.9. GSH Assay

The hepatic GSH contents were assayed using a commercially available GSH assay kit. After reaction with 5,5-dithiobes-(2-ni-trobenzoic acid) (DTNB), the GSH contents were measured by a spectrophotometer (Shimadzu UV-2501PC) at 405 nm. The GSH contents were calculated as the contents (μmol GSH) per mg protein.

### 4.10. SOD1 Activity Assay

SOD1 activity was determined following the method previously described [18]. SOD1 activities were calculated as units per mg protein.

### 4.11. CAT Activity Assay

CAT activity was determined as described in our previous work [13]. CAT activity was presented as μM H_2_O_2_ consumed/min/mg of tissue protein.

### 4.12. Immunoprecipitation

Immunoprecipitations were conducted as previously described [16]. After preclearing with protein A-sepharose beads for 1 h at 4 °C, 500 μg protein were incubated with 3 μg anti-RIP2 antibodies overnight at 4 °C. Immune complexes were precipitated by protein A-sepharose beads. Following washing six times with 25 mM HEPES buffer, pH 7.4 containing 10 mM MgCl_2_, 1 mM NaF, 1% NP-40, and 1 mM Na_3_VO_4_, immune complexes were analyzed by Western blot with anti-NOD1, anti-NOD2 and anti-RIP2 antibodies.

### 4.13. Western Blot

The Western blot analyses were performed as previously described [13,18]. Briefly, protein samples were separated by electrophoresis on denaturing SDS-PAGE gels and then transferred to polyvinylidene difluoride (PVDF) membranes (Roche Diagnostics Corporation, Indianapolis, IN, USA). After blocked with 5% non-fat milk in 0.1% Tween-20/TBS, the membrane was incubated with primary antibodies overnight at 4 °C. Then the membranes were incubated with HRP-conjugated secondary antibodies and were visualized by 20× LumiGLO^®^ Reagent and 20× peroxide. The optical density (OD) values of the detected bands were quantified by Scion Image analysis software (Scion Corp., Frederick, MD, USA) and were normalized using appropriate internal controls (optical density_detected protein_/optical density_internal control_).

### 4.14. Quantitative Real Time Polymerase Chain Reaction

The quantitative real time polymerase chain reaction was performed as described in our previous work [18]. The primers used were: *PEPCK*, Forward: 5′-CAGCCAGTGCCCCATTATT-3′, Reverse: 5′-CCACCAAAGATGATACCCTCA-3′; *G6P*, Forward: 5′-TGGCCTGGCTTATTGTACCT-3′, Reverse: 5′-GTGCTAAGAGGAAGACCCGA-3′; *NOD1*, Forward: 5′-TGACGTTCCTGGGTTTATACAACA-3′, Reverse: 5′-CCAGGATTTGGGCCACATAC-3′; *NOD2*, Forward: 5′-CCTGGTACGTGCCCAAAGTAG-3′, Reverse: 5′-GCCAAGTAGAAAGCGGCAAA-3′; *IL-1β*, Forward: 5′-AAATACCTGTGGCCTTGGGC-3′, Reverse: 5′-CTTGGGATCCACACTCTCCAG-3′; *TNF-α*, Forward: 5′-TCTCATTCCTGCTTGTGG-3′, Reverse: 5′-ACTTGGTGGTTTGCTACG-3′; *MCP-1*, Forward: 5′-AGGTCCCTGTCATGCTTCTG-3′, Reverse: 5′-GCTGCTGGTGATCCTCTTGT-3′; *β-actin*, Forward: 5′-TGCTGTCCCTGTATGCCTCTG-3′, Reverse: 5′-TTGATGTCACGCACGATTTCC-3′. The relative levels of target mRNAs, were normalized to β-actin mRNA, and were calculated by the comparative cycle threshold (Ct) method.

### 4.15. Statistical Analysis

Data analysis was conducted using SPSS software version 11.5 (SPSS Inc., Chicago, IL, USA). All of the data were expressed as the means ± standard deviation (SD) and were analyzed by one-way ANOVA followed by Tukey’s Honestly Significant Difference (HSD) post-hoc test and Student’s *t*-test. Statistical significance was set to *p* < 0.05.

## 5. Conclusions

Troxerutin displayed significant inhibitory effects on the enhancement of hepatic gluconeogenesis by attenuating ER stress-mediated NOD activation and consequent inflammation in the livers of HFD-treated mice, which might be mediated by its anti-obesity effect. Our results showed that troxerutin abated oxidative stress to restrain ER stress, therefore blocking NOD signaling, consequently suppressing NF-κB p65 transcriptional activation and release of inflammatory cytokines, thereby diminishing hepatic gluconeogenesis, ultimately lowering fasting glucose levels in HFD-fed mice. Moreover, troxerutin ameliorated obesity and decline of circulating adiponectin in HFD-treated mouse, indicating these improvements of hepatic glucose disorders might be mediated by its anti-obesity effect. This study provides insight into a novel mechanism for T2D pathogenesis and indicates that troxerutin is a candidate for pharmacological treatment of fasting hyperglycemia via its inhibitory effects on elevated hepatic gluconeogenesis.
